# Modeling the LPS Neutralization Activity of Anti-Endotoxins

**DOI:** 10.3390/molecules14051869

**Published:** 2009-05-20

**Authors:** Chadinee Thippakorn, Thummaruk Suksrichavalit, Chanin Nantasenamat, Tanawut Tantimongcolwat, Chartchalerm Isarankura-Na-Ayudhya, Thanakorn Naenna

**Affiliations:** 1 Department of Clinical Microbiology, Faculty of Medical Technology, Mahidol University, Bangkok 10700, Thailand; 2 Department of Industrial Engineering, Faculty of Engineering, Mahidol University, Nakhon Pathom 73170, Thailand

**Keywords:** lipopolysaccharide, endotoxin, anti-endotoxin, artificial neural network, QSAR

## Abstract

Bacterial lipopolysaccharides (LPS), also known as endotoxins, are major structural components of the outer membrane of Gram-negative bacteria that serve as a barrier and protective shield between them and their surrounding environment. LPS is considered to be a major virulence factor as it strongly stimulates the secretion of pro-inflammatory cytokines which mediate the host immune response and culminating in septic shock. Quantitative structure-activity relationship studies of the LPS neutralization activities of anti-endotoxins were performed using charge and quantum chemical descriptors. Artificial neural network implementing the back-propagation algorithm was selected for the multivariate analysis. The predicted activities from leave-one-out cross-validation were well correlated with the experimental values as observed from the correlation coefficient and root mean square error of 0.930 and 0.162, respectively. Similarly, the external testing set also yielded good predictivity with correlation coefficient and root mean square error of 0.983 and 0.130. The model holds great potential for the rational design of novel and robust compounds with enhanced neutralization activity.

## Introduction

Lipopolysaccharides (LPS) are major structural components of the outer membrane of Gram-negative bacteria. LPS confers a net negative charge to the membrane and thereby serves as a protective shield against antibacterial agents, as well as playing a crucial role in maintaining the integrity of the overall membrane structure. LPS are endotoxins which when present in the systemic circulation lead to the development of septic shock. The endotoxins initiate such events by first binding to CD14/TLR4/MD2 receptor complex on phagocytic cells causing the release of pro-inflammatory cytokines which consequently increase the releasing of other inflammatory mediators in many cell types (e.g. neutrophils, monocytes, vascular endothelial cell) as well as initiate the neuroendocrine response. Aside from this, LPS can also mediate the activation of plasma protein cascades (e.g. complement system) [1,2,3,4]. Among the mediators produced by activated macrophage, tumor necrosis factor (TNF) is the most important and the earliest to be released. TNF can amplify the response to endotoxins by further activating inflammatory cytokines (e.g. IL-1, IL-6, and IL-8) which consequently leads to the synthesis of other mediators such as arachidonic acid, platelet-activating factor (PAF), nitric oxide and reactive oxygen species [5,6]. Such elevated levels of mediator decrease blood flow to vital organs (e.g. kidney, heart, and brain) by increasing vascular permeability as well as causing microvacular occlusion and damage thereby leading to multiple organ dysfunctions and eventually culminating in death [7].

Among those susceptible to the development of sepsis are children, immunocompromised individuals, and the elderly. In fact, global incidences of septic shock have increased over the past decade as a result of the growing number of immunologically compromised patients. Furthermore, LPS is considered to be one of the leading causes of mortality in intensive care units worldwide [8,9,10]. Therapeutic strategies for increasing the chances of survival include: (i) modulating inflammatory mediator release via the use of anti-cytokine or anti-inflammatory agents, (ii) supporting major organ dysfunction by increasing blood flow and (iii) administrating early antibiotic or anti-endotoxin usage. Therefore, drugs or compounds able to combat LPS toxicity have been of great interest [11]. Potential therapeutic agents such as anti-endotoxin antibodies, short peptides and lipopolyamines, as well as other small molecules, have been found to display promising sequestering effect towards lipid A but the efficiency of those compounds needs to be improved [12,13,14,15,16,17,18].

The toxic compartment of LPS is their structurally conserved glycolipid component called lipid A. Lipid A is a fatty acid chain bound to two phosphorylated glucosamine residues that is linked to the oligosaccharide core and the distal O-antigen polysaccharide chain. The O-polysaccharide chain is made of oligosaccharide repetitive units (O-units) while the oligosaccharide core is made of an inner core (ketodeoxyoctonic acid and heptose) and an outer core. The diversity in the composition and length of the O-antigen vary among different bacterial species.

In order to investigate the molecular parameters of interaction between lipid A and anti-endotoxin agents, Guo *et al*. utilized molecular modeling analysis for elucidating the underlying mechanism governing such high binding affinity. Their results indicated that a correlation exists between the binding affinity and the electrostatic interaction of ligands with lipid A [19]. In parallel, our previous investigations have revealed that quantitative structure-activity/property relationship (QSAR/QSPR) are useful tools for establishing relationships between molecular structures with the respective activities or properties of a wide array of biological and chemical systems [20,21,22,23,24,25,26]. Elucidations on the feasibility of potential ligands prior to performing actual experimentations are useful on the economic and time-saving view points.

To the best of our knowledge, we present the first development of a quantitative structure-activity relationship model of LPS neutralization activity by anti-endotoxins as modeled by multivariate analysis. Molecular descriptors accounting for charge and electronic properties of anti-endotoxins were used as input variables for calculating the half-maximal effective displacement (ED_50_).

## Results and Discussion

### Structural considerations

It has been reported that electrostatic and hydrophobic forces are necessary for LPS neutralization activity [27,28,29,30]. To eliminate the endotoxin, an agent would need to bind to lipid A. This is achieved by small molecules that are capable of engaging in strong hydrogen bond formation with the cationic and phosphate groups of lipid A. Furthermore, hydrophobic moieties are also necessary in stabilizing and enhancing the affinity of the agent in binding to the endotoxin [31]. Therefore to account for the electrostatic and hydrophobic forces, descriptors derived from RECON and Spartan’04 software packages were used for QSAR model development. Descriptors from RECON, which is based on the TAE methodology, are suitable for this study as it directly accounts for the molecular recognition in terms of the electronic charge properties. Additional descriptors from Spartan’04 were selected to account for the aforementioned interaction forces. Particularly, the hydrophobic interaction was approximated by the water accessible hydrophobic surface area of the molecule (CPK_Area_). Molecular descriptors such as total energy (*E_Total_*), atomic charge (*Q_A_*) and total hydrogen atomic charge (*Q_TH+_*) are important auxiliary variables that could also account for the electrostatic properties of the molecules. It was observed that the combination of both sets of descriptors exerted a dramatic enhancement of predictive power than solely relying on TAE descriptors (data not shown).

### Variable reduction

**Figure 1:**
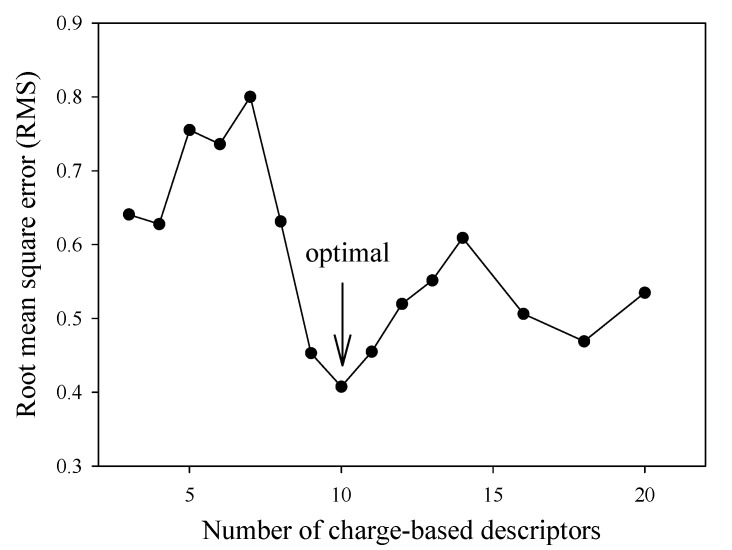
Plot of RMS as a function of the number of TAE molecular descriptors.

Prior to performing the calculation of LPS neutralization activity, the redundant and multi-collinear descriptors present in the dataset were initially reduced by UFS in order to achieve a better efficiency of prediction. The subset of descriptors left after variable reduction was in the range of 3 and 20. The optimal number of descriptor to use was determined by making a plot of the number of selected descriptors as a function of RMS (Figure 1). It was observed that the optimal number was 10, which was selected for further investigations.

### Parameter optimization

The network architecture was determined by a trial-and-error adjustment of various parameters for the purpose of obtaining an optimal configuration. The empirically determined parameters included the number of nodes in the hidden layer, the learning epoch size, and the learning rate and momentum. Parameter that exhibited the lowest RMS was chosen as optimum. The optimal number of hidden nodes was determined by varying the number of nodes from 1 to 25.

**Figure 2:**
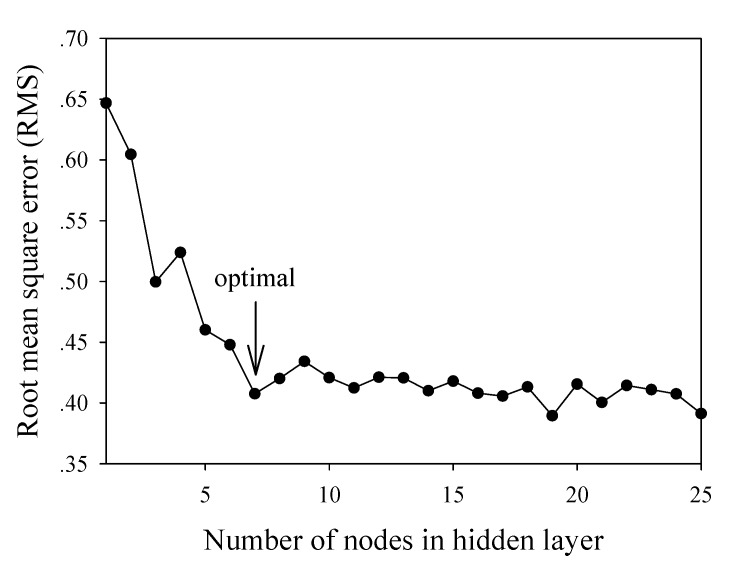
Plot of RMS as a function of the number of nodes in hidden layer.

**Figure 3:**
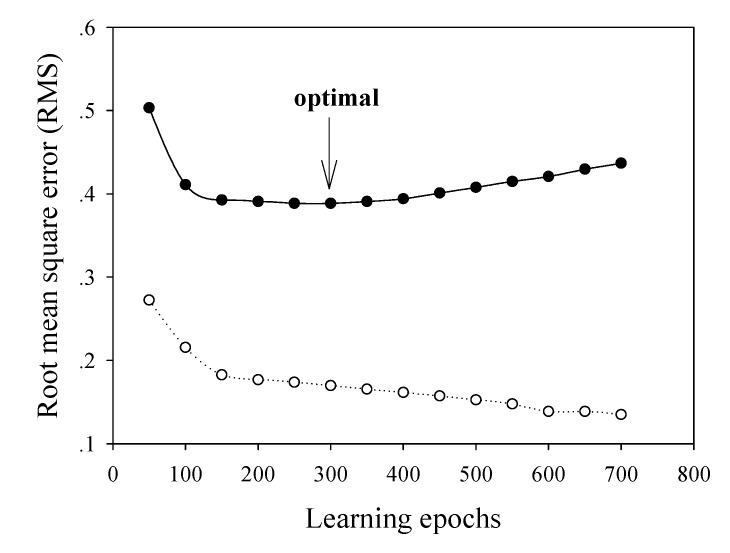
Plot of RMS as a function of the number of learning epochs. The cross-validated test set is represented as a solid line while the training set is represented as a dotted line.

As represented in the plot of RMS versus hidden nodes (Figure 2), the optimal number of nodes was found to be seven. In order to ovoid overtraining of the network, the learning epoch size was subsequently optimized from 1 to 700 in increments of 50 and learning was stopped once a detectable rise in RMS for the leave-one-out cross-validated testing set was observed. The best learning time could be observed by making a plot of the RMS as a function of the learning epoch size (Figure 3). The optimal value was found to be 300.

**Figure 4:**
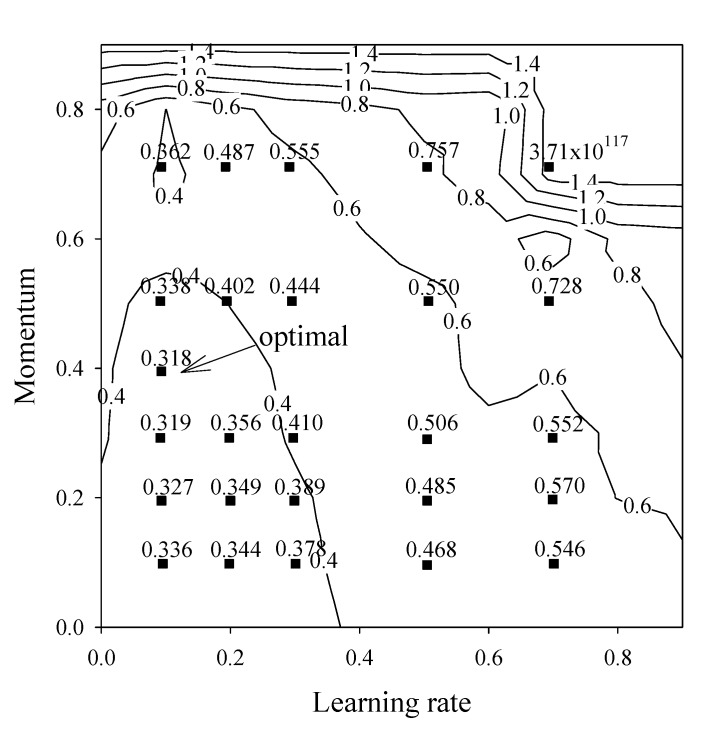
Contour plot of RMS as function of learning rate and momentum for the cross-validated testing set.

Subsequently, the optimal learning rate and momentum was selected by making a contour plot of the RMS as function of the learning rate and momentum (Figure 4). The lines in the contour plot represented constant values of RMS, while shaded boxes designated RMS values that were obtained from the learning procedures and fitted onto the same surface model [32]. As shown on the contour plot, the best learning rate and momentum lies in the middle left region of the graph and was found to be 0.1 and 0.4, respectively.

### Prediction of LPS neutralization activity using artificial neural network

**Figure 5:**
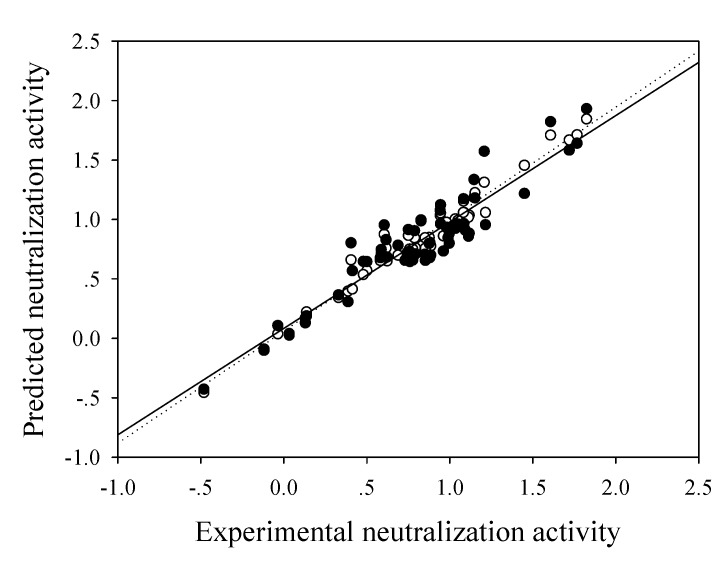
Plot of predicted versus experimental ED_50_ for training set (○; regression line is represented as a dotted line) and leave-one-out cross-validation testing set (●; regression line is represented as a solid line) of anti-endotoxins.

The optimal configuration of the predictive model of LPS neutralization activity was identified to be 7, 300, 0.1 and 0.4 for the number of hidden nodes, learning epoch size, learning rate and momentum, respectively. The network was created by means of a leave-one-out cross validation [33,34,35], by which one sample of the dataset was withdrawn for use as the test set while the rest served as the training set. This process was repeated iteratively until all samples of the dataset were used as the test set [32,36,37]. It was observed that the predicted and experimental neutralization activities were moderately correlated as can be observed from the correlation coefficient of 0.781. To further refine the model, identification of potential outliers present inherently in the predictive model was performed using standard statistical analysis where compounds with absolute standardized residuals exceeding the cut-off value of 2 were marked as outliers. This statistical analysis was reiterated to yield a final predictive model which was constructed using previously determined optimal set of network parameters. As indicated in Table 1, results yielded correlation coefficient and root mean square error of 0.980 and 0.124 for the training set, respectively, while 0.930 and 0.162 was observed for the testing set. A plot showing the experimental versus predicted LPS neutralization activity for model 6 is shown in Figure 5.

**Table 1:** Summary of the predictive performance.

Model	*N*	*r*_Tr_	RMS_Tr_	*r*_CV_	RMS_CV_	*R^2^*	*R^2^_adj_*	*F* ratio	Critical *F* value
1	73	0.938	0.167	0.781	0.287	0.610	0.524	6.480	1.866^a^
2	69	0.942	0.158	0.728	0.341	0.530	0.419	4.350	1.879^b^
3	64	0.947	0.141	0.857	0.221	0.734	0.665	9.658	1.899^c^
4	63	0.952	0.146	0.823	0.250	0.677	0.591	7.186	1.904^d^
5	60	0.972	0.124	0.907	0.186	0.823	0.773	14.946	1.918^e^
6	58	0.980	0.124	0.930	0.162	0.865	0.825	19.680	1.929^f^

*N*, sample size of the data set; *r*_Tr_, correlation coefficient of training set; RMS_Tr_, root mean square error of training set; *r*_CV_, correlation coefficient of leave-one-out cross validation testing set; RMS_CV_, root mean square error of testing set; *R^2^*, squared correlation coefficient of leave-one-out cross validation testing set, *R^2^_adj_*, adjusted squared correlation coefficient of leave-one-out cross validation testing set; *F* ratio, calculated *F* ratio of cross validation testing set. Critical *F* values at the 95% confidence level with *m* and *n* – *m* – 1 degrees of freedom (*F*_(M__、__n-__m-__1)_) as follows ^a^ *F*_(14.58)_, ^b^ *F*_(14.54)_, ^c^ *F*_(14.49)_, ^d^ *F*_(14.48)_, ^e^ *F*_(14.45)_, and ^f^ *F*_(14.43)_ [38].

**Figure 6:**
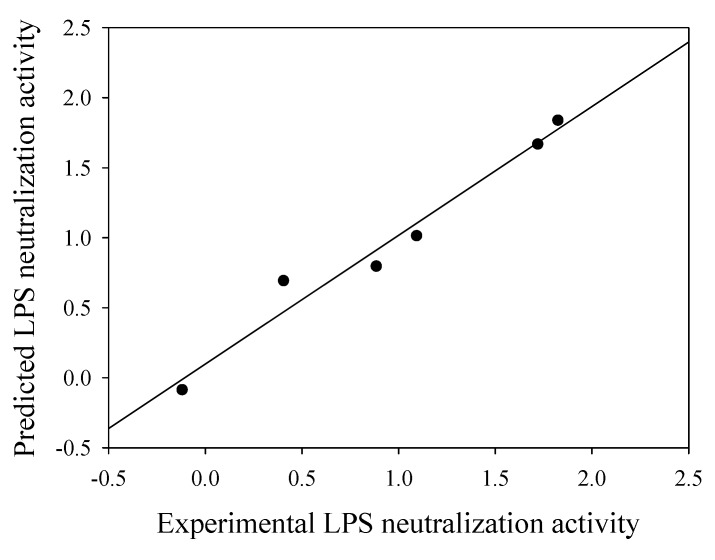
Plot of predicted versus experimental ED_50_ for the leave-one-out cross-validation testing set of anti-endotoxins.

In order to evaluate the predictive power of the QSAR model discussed herein, an external testing set would be necessary. To achieve this, the data set from model 6 was further divided into two sets of data: (i) one portion for deriving the optimal network parameters by leave-one-out cross-validation and (ii) an external testing set for evaluating the extrapolation capability of the QSAR model. This was carried out by randomly selecting 10% of the data set as an external testing set which equates to 6 data samples while the remaining 52 data samples were used for performing leave-one-out cross-validation. Optimization of the network parameter was performed as previously discussed to give the optimal parameters as follows: 15 nodes in the hidden layer, 150 learning epochs, learning rate of 0.2 and momentum of 0.4. This set of parameter was used for deriving the predictive performance of the external test set. Results indicated that the proposed QSAR model could accurately predict the LPS neutralization activity as observed by the correlation coefficient of 0.983 and root mean square error of 0.130 as shown in Figure 6.

## Conclusions

The LPS neutralization activities of anti-endotoxin agents were modeled using back-propagation neural network. The predicted neutralization activities were found to be in good agreement with the experimental values. Analysis of these results suggests that the use of charge-based descriptors and quantum chemical descriptors were useful and necessary in obtaining good prediction that is representative of the experimental values. Therefore, such methodology proposed in this study demonstrates a facile approach for the design of novel anti-endotoxin agents with robust properties.

## Experimental

### Data collection

The half-maximal effective displacement (ED_50_) of 80 anti-endotoxins were collected from the literature [27,31,40] (Table 1). Seven compounds were identified as inactive and were removed due to their high level of ED_50_ >200.

### Descriptor generation

The two-dimensional structure of each compound was drawn in ChemAxon’s MarvinSketch [41] and exported as SMILES notation. The three-dimensional molecular structures were constructed using the molecular building module of Spartan’04 [42] and subsequently submitted for calculation of quantum chemical descriptors. Likewise, the SMILES notation served as input for the calculation of charge-based descriptors by RECON.

RECON (version 5.5) was used for the generation of 248 transferable atom equivalent (TAE) molecular descriptors. The TAE methodology, based on Bader’s quantum theory of atoms in molecule, was developed by Brenemen and co-workers for the rapid reconstruction of molecular charge density and molecular electronic property via pre-computed *ab initio* atomic charge density fragment [42]. TAE descriptors were selected for the prediction of LPS neutralization activity as they could account for the electronic properties of molecules, which is crucial for modeling molecular interaction [25] of the anti-endotoxins with their respective LPS target.

Aside from electronic properties, the involvement of hydrophobic and electrophilic forces has also been demonstrated to play a crucial role in the biological activity of the anti-endotoxins [11,12]. Hence, Spartan’04 was employed for generation of the following quantum chemical descriptors: molecular weight (MW), water accessible hydrophobic surface area of the molecule (CPK_Area_), total energy (*E_Total_*), and atomic charge (*Q_A_*). The three-dimensional structures of each anti-endotoxin compound were initially optimized using the Merck Molecular Force Field (MMFF) in conjunction with Monte Carlo simulation or systematic conformational search to identify the lowest energy geometry. These pre-optimized structures were then calculated at the semi-empirical level using the Parameterization Method 3 (PM3) [42].

An additional descriptor, the total hydrogen atomic charge (*Q_TH+_*) was calculated according to the following equation:

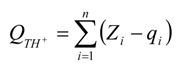
(1)
where *Z_i_, q_i_* and n represent the atomic numbers, the atomic electron populations and the number of hydrogen atoms, respectively.

### Descriptor reduction

The molecular descriptors were subjected to variable reduction in order to reduce computational time, minimize multi-collinearity and eliminate redundancy of the descriptors. This was performed using UFS, version 1.8, which is a computer program based on the Unsupervised Forward Selection (UFS) algorithm [43]. Briefly, UFS removes variables when the standard deviation is less than the predefined sdevmin and terminates when the squared multiple correlation coefficients of the remaining variables exhibit values greater than the r-squared-max (*R^2^_max_*). The standard deviation was left as default at 0.0005 while the *R^2^_max_* was varied between 0 and 0.99. Thus, 248 descriptors were reduced to a range of three to 20 descriptors.

**Table 2:** Data set of the anti-endotoxins^a^.

No.		Compound Name			Exp. ED_50_			Exp. logED_50_			Pred. logED_50_		Residual	
1^c^		N^1^-Acetyl-1,16-diamino-4,8,13triazahexadecane tetrakis(trifluoroacetic acid)			107.450			2.031			1.395		0.636	
2		N^1^-Nonanoyl-1,16-diamino-4,8,13-triazahexadecane tetrakis(trifluoroacetic acid)			0.920			-0.036			0.254		-0.290	
3		N^1^-Decanoyl-1,16-diamino-4,8,13-triazahexadecane tetrakis(trifluoroacetic acid)			1.080			0.033			-0.210		0.243	
4		N^1^-Pentadecanoyl-1,16-diamino-4,8,13-triazahexadecane tetrakis(trifluoroacetic acid)			1.350			0.130			0.118		0.012	
5		N^1^-Heptadecanoyl-1,16-diamino-4,8,13-triazahexadecane tetrakis(trifluoroacetic acid)			1.370			0.137			0.335		-0.198	
6		N^1^-Nonadecanoyl-1,16-diamino-4,8,13-triazahexadecane tetrakis(trifluoroacetic acid)			2.440			0.387			0.306		0.081	
7		N^1^,N^20^-Dinonanoyl-1,20-diamino-4,8,13,17-tetrazaicosane tetrakis(trifluoroacetic acid)			0.330			-0.481			-0.203		-0.278	
8		N^1^,N^20^-Didecanoyl-1,20-diamino-4,8,13,17-tetrazaicosane tetrakis(trifluoroacetic acid)			0.760			-0.119			0.058		-0.177	
9^f^		N^1^,N^20^-Didodecanoyl-1,20-diamino-4,8,13,17-tetrazaicosane tetrakis(trifluoroacetic acid)			6.870			0.837			0.132		0.705	
10^f^		N^1^,N^20^-Dipentdecanoyl-1,20-diamino-4,8,13,17-tetrazaicosane tetrakis(trifluoroacetic acid)			8.670			0.938			1.518		-0.580	
11		N^1^,N^20^-Diheptadecanoyl-1,20-diamino-4,8,13,17-tetrazaicosane tetrakis (trifluoroacetic acid)			52.530			1.72			1.806		-0.086	
12		N^1^,N^20^-Dinonadecanoyl-1,20-diamino-4,8,13,17-tetrazaicosane tetrakis(trifluoroacetic acid)			66.730			1.824			1.658		0.166	
13		(S)-1-(1-(2-(2-aminoethoxy)ethylamino)-1-oxo-3-phenylpropan-2-yl)-3-octadecylurea			12.400			1.093			0.871		0.222	
14		(S)-N-(2-(2-aminoethoxy)ethyl)-2-(2 (octadecylamino)acetamido)-3-phenylpropanamide			2.540			0.405			0.706		-0.301	
15		(S)-N-(2-(2-aminoethoxy)ethyl)-2-(3-(octadecylamino)propanamido)-3-phenylpropanamide			7.680			0.885			0.591		0.294	
16		(S)-1-(1-(2-(2-aminoethoxy)ethylamino)-3-(1H-imidazol-4-yl)-1-oxopropan-2-yl)-3-octadecylurea			13.100			1.117			0.881		0.236	
17		(S)-N-(2-(2-aminoethoxy)ethyl)-3-(1H-imidazol-4-yl)-2-(2-(octadecylamino)acetamido)propanamide			3.170			0.501			0.557		-0.056	
18		(S)-N-(2-(2-aminoethoxy)ethyl)-3-(1H-imidazol-4-yl)-2-(3-(octadecylamino)propanamido)propanamide			5.380			0.731			0.869		-0.138	
19		1-(2-(2-(2-aminoethoxy)ethylamino)-2-oxoethyl)-3-octadecylurea			14.000			1.146			0.980		0.166	
20		N-(2-(2-aminoethoxy)ethyl)-2-(2 (octadecylamino)acetamido)acetamide			14.200			1.152			1.187		-0.035	
21		N-(2-(2-(2-aminoethoxy)ethylamino)-2-oxoethyl)-3-(nonadecylamino)propanamide			10.800			1.033			1.025		0.009	
22		(S)-1-(1-(3-aminopropylamino)-1-oxo-3-phenylpropan-2-yl)-3-octadecylurea			8.800			0.944			0.990		-0.046	
23		(S)-N-(3-aminopropyl)-2-(2-(octadecylamino)acetamido)-3-phenylpropanamide			4.130			0.616			0.707		-0.091	
24		(S)-N-(3-aminopropyl)-2-(3-(octadecylamino)propanamido)-3-phenylpropanamide			5.750			0.760			0.666		0.094	
25		(S)-1-(1-(3-aminopropylamino)-3-(1H-imidazol-4-yl)-1-oxopropan-2-yl)-3-octadecylurea			4.870			0.688			0.814		-0.126	
26^f^		(S)-N-(3-aminopropyl)-3-(1H-imidazol-4-yl)-2-(2-(octadecylamino)acetamido)propanamide			6.860			0.836			0.414		0.422	
27		(S)-N-(3-aminopropyl)-3-(1H-imidazol-4-yl)-2-(3-(octadecylamino)propanamido)propanamide			3.010			0.479			0.994		-0.515	
28^c^		1-(2-(3-aminopropylamino)-2-oxoethyl)-3-octadecylurea			6.610			0.82			1.493		-0.673	
29^b^		N-(3-aminopropyl)-2-(2-(octadecylamino)acetamido)acetamide			2420			3.384			-		-	
30		N-(2-(3-aminopropylamino)-2-oxoethyl)-3-(octadecylamino)propanamide			6.140			0.788			0.961		-0.173	
31^b^		(S)-N-(5-aminopentyl)-2-(2-(octadecylamino)acetamido)-3-phenylpropanamide			3850			3.585			-		-	
32		(S)-N-(5-aminopentyl)-2-(3-(octadecylamino)propanamido)-3-phenylpropanamide			7.510			0.876			0.861		0.015	
33		(S)-1-(1-(5-aminopentylamino)-3-(1H-imidazol-4-yl)-1-oxopropan-2-yl)-3-octadecylurea			12.100			1.083			1.343		-0.260	
34^g^		(S)-N-(5-aminopentyl)-3-(1H-imidazol-4-yl)-2-(3-(octadecylamino)propanamido)propanamide			18.700			1.272			0.702		0.570	
35		1-(2-(5-aminopentylamino)-2-oxoethyl)-3-octadecylurea			28.200			1.450			0.944		0.506	
36		N-(5-aminopentyl)-2-(2-(octadecylamino)acetamido)acetamide			11.200			1.049			0.967		0.082	
37		N-(2-(5-aminopentylamino)-2-oxoethyl)-3-(octadecylamino)propanamide			9.770			0.990			0.900		0.090	
38^g^		1-(3-aminopropyl)-3-octadecylurea			3.800			0.580			0.839		-0.259	
39		N-(3-aminopropyl)-2-(octadecylamino)acetamide			9.920			0.997			0.652		0.346	
40		N-(3-aminopropyl)-3-(octadecylamino)propanamide			6.210			0.793			0.794		-0.001	
41		1-(5-aminopentyl)-3-octadecylurea			8.740			0.942			0.841		0.101	
42		1-(2-(2-aminoethoxy)ethyl)-3-octadecylurea			12.150			1.085			1.032		0.053	
43		N-(5-aminopentyl)-2-(octadecylamino)acetamide			4.030			0.605			0.908		-0.303	
44		N-(2-(2-aminoethoxy)ethyl)-2-(octadecylamino)acetamide			9.160			0.962			0.797		0.165	
45		N-(5-aminopentyl)-3-(octadecylamino)propanamide			7.610			0.881			0.895		-0.014	
46		N-(2-(2-aminoethoxy)ethyl)-3-(octadecylamino)propanamide			5.730			0.758			0.883		-0.125	
47		L-Lys-N1-spermine			40.420			1.607			1.725		-0.118	
48		D-Lys-N1-spermine			58.420			1.767			1.611		0.157	
49^e^		L-Lys-ε-(eicosanoyl)-N1-spermine			6.460			0.810			1.052		-0.242	
50		D-Lys-ε-(stearoyl)-N1-spermine			8.800			0.944			0.988		-0.044	
51		L-Lys-ε-(stearoyl)-N1-spermine			16.390			1.215			0.888		0.327	
52		L-Lys(ene-Δ11-stearoyl)-N1-spermine			4.200			0.623			0.812		-0.189	
53		L-Lys-ε-(heptadecanoyl)-N1-spermine			6.710			0.827			0.990		-0.163	
54^c^		L-Lys-ε-(hexadecanesulfonamide)-N1-spermine			5.930			0.773			1.277		-0.504	
55		D-Lys-ε-(palmitoyl)-N1-spermine			9.940			0.997			0.926		0.071	
56		L-Lys(palmitoyl)-N1-spermine			10.740			1.031			0.962		0.069	
57		L-Lys(ene-Δ9-palmitoyl)-N1-spermine			3.820			0.582			0.494		0.088	
58		L-Lys-ε-(myristoyl)-N1-spermine			5.630			0.751			0.986		-0.235	
59		L-Lys-ε-(octanoyl)-N1-spermine			12.970			1.113			1.112		0.002	
60^b^		D-Lys-ε-(isopropyl)-N1-spermine			298.850			2.475			-		-	
61^b^		D-Lys-ε-(dimethylpropyl)-N1-spermine			327.040			2.515			-		-	
62		D-Lys-ε-(2-norbornaneacetoyl)-N1-spermine			16.160			1.208			0.915		0.293	
63^c^		D-Lys-ε-(4-biphenycarboxamide)-N1-spermine			7.860			0.895			1.554		-0.659	
64		L-Lys-ε-(4-(1-pyrene)-butanoyl)-N1-spermine			7.090			0.851			0.296		0.555	
65^b^		L-Lys-ε-(methylpolyethyleneglycolpropionyl)-N1-spermine			310.950			2.493			-		-	
66^b^		L-Lys-ε-(2-[2-(2-methoxyethoxy)ethoxy]acetoyl)-N1-spermine			572.500			2.758			-		-	
67^b^		L-Lys-ε-(2-(2-methoxyethoxy)acetoyl)-N1-spermine			495.190			2.695			-		-	
68		L-Lys-ε-(hexadecyl)-N1-spermine			5.560			0.745			0.619		0.126	
69		L-Lys-ε-(ene -Δ11^-^hexadecyl)-N1-spermine			2.590			0.413			-0.049		0.462	
70		D-Lys-ε-(n-heptyl)-N1-spermine			3.860			0.587			0.751		-0.164	
71		L-Lys-ε-(n-heptyl)-N1-spermine			5.990			0.777			0.696		0.081	
72		L-Lys-ε-(bis-(n-heptyl))-N1-spermine			2.140			0.330			0.355		-0.025	
73		L-Lys-ε-(n-hexyl)-N1-spermine			7.130			0.853			0.877		-0.024	
74		D-(S)-Lys-ε-(ene-Δ6(3,7-dimethyl-1-octyl))-N1-spermine			9.550			0.980			0.951		0.029	
75		L-Lys-ε-(3,3-dimethyl-1-butyl)-N1-spermine			12.070			1.082			1.174		-0.092	
76^d^		D-Lys-ε-(3,3-dimethyl-1-butyl)-N1-spermine			10.930			1.039			1.244		-0.205	
77^d^		D-Lys-ε-(3-methylpropyl)-N1-spermine			100.580			2.003			1.385		0.618	
78^d^		L-(R)-Lys-ε-((2-isoproply-5-methyl)cyclohexyl)-N1-spermine			16.080			1.206			1.280		-0.074	
79^d^		L-Lys-ε-(bis-(cyclohexyl))-N1-spermine			4.040			0.606			0.099		0.507	
80^d^		D-Lys-ε-(4-phenylbenzyl)-N1-spermine			3.710			0.569			0.056		0.513	

^a^ Compounds no. 1-12, 13-46 and 47-80 were derived from [39], [31] and [27], respectively; ^b^ Compounds identified as inactive and removed from data set; Compounds identified as outliers in Models ^c^ 1, ^d^ 2, ^e^ 3, ^f^ 4, and ^g^ 5 according to standardized residual cut-off of 2.

### Overview of artificial neural network

Artificial neural network (ANN) is a computational model that mimics the learning process of the human brain. This is performed in a supervised manner where the model attempts to find a relationship between the independent and dependent variables. ANN is comprised of multiple layers of artificial neurons that are interconnected in a feed-forward manner where signals are relayed from the input layer through the hidden layer and finally to the output layer. The connections among the network of neurons are assigned numerical values known as weights which alter the signal flow through the neural network and governing its predictive performance. Particularly, the weights are adjusted adaptively to reduce the error by back-propagating signals from the output layer back to the input layer through the hidden layer. Readers are referred to the excellent book by Zupan and Gasteiger [32] for further methodological information.

### Prediction of LPS neutralization activity

The predictive model of LPS neutralization activity was developed using back-propagation neural network as calculated by Weka, version 3.4.3 [44]. The independent variables, comprising of TAE descriptors and quantum chemical descriptors, of each compound were normalized to a range of 0 and 1 according to the following equation:

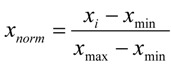
(2)
where *x_norm_*, *x_i_*, *x_min_*, *x_max_* represent the normalized data, the value of each instance, the minimum value, and the maximum value of the dataset, respectively.

The optimal neural network parameters were obtained by an empirical trial-and-error search of the number of nodes in the hidden layer, the learning epoch size, the learning rate (*η*) and momentum (*μ*) constant. A learning epoch refers to a complete cycle of data propagated through the layers of the network in a feed-forward manner. *η* controls the speed of weight adjustment, while *μ* prevents sudden changes in attaining the solution. Under an incremental tuning of such parameter, root mean square error (RMS) was simultaneously measured as an indicator of predictive error, which is calculated according to the following equation:

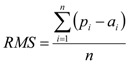
(3)
where *p_i_*, *a_i_*, and *n* represent the predicted output, the actual output and the number of compounds in the dataset, respectively. As each neural network calculation operates via random initialization of the weights, the averaged RMS of ten runs was used as a measure of predictive error by adjusting the random seed from 0 to 9.

### Internal validation procedure

Generation of training and testing sets was made using leave-one-out cross-validation (LOO-CV). Briefly, LOO-CV involved the leaving out of one molecule as the testing set and using the remaining as training set. This is performed iteratively until all samples were given the chance to be left out as testing sets.

### Statistical analysis

The adjusted *R^2^* takes into consideration such information as the number of independent variables that are present in the predictive model. This is calculated according to the following equation:

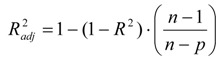
(4)
where *n* is the sample size and *p* is the number of independent variables.

The *F* ratio measures the explained variance (*R^2^*) in relation to the unexplained variance (1*– R^2^*) with *m* and *n*-*m*-1 degrees of freedom:

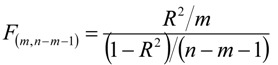
(5)
where *m* is the number of independent variables and *n* is the number of compounds presented in the data set.

Outlying molecules were identified by standardization of the residuals according to the following equation:

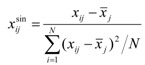
(6)
where *x*^sin^_*ij*_ represents the standardized residual, *x_ij_* represents the residual of each sample, 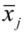 represents the mean of the residual, and *N* represents the sample size of the data set. The cut-off value for the absolute standardized residual was set to 2.
